# Effect of transcranialdirect current stimulation on the right brain temporal area on processing approach and avoidance attitudes with negation

**DOI:** 10.3389/fnhum.2022.971051

**Published:** 2022-11-10

**Authors:** Aarón Nuez, Iván Padrón, Cristian Reyes-Moreno, Hipólito Marrero

**Affiliations:** ^1^Facultad de Humanidades y Ciencias Sociales, Psicología, Universidad Tecnológica del Perú, Lima, Peru; ^2^Instituto Universitario de Neurociencias, Universidad de La Laguna, Santa Cruz de Tenerife, Spain; ^3^Departamento de Psicología Evolutiva y de la Educación, Universidad de La Laguna, San Cristóbal de La Laguna, Spain; ^4^Experimental Psychology Lab, Department of Psychology, Carl von Ossietzky University Oldenburg, Oldenburg, Germany; ^5^Departamento de Psicología Cognitiva, Social y Organizacional, Universidad de La Laguna, San Cristóbal de La Laguna, Spain

**Keywords:** approach/avoidance intentionality, attitudes, tDCS, action understanding, reading, negation, superior temporal sulcus

## Abstract

Language describes approach/avoidance intentionality by means of attitudinal verbs (e.g., accept vs. reject). The right superior temporal sulcus (rSTS) has been shown to be recruited in processing action goals and approach intentionality in social contexts. In this study, we examine whether transcranial direct current stimulation (tDCS) of this area improves the processing of attitudinal verbs (either of approach or avoidance) in the context of affirmative and negative sentences [e.g., Julio (did not)/included meat on the grocery list]. After being subjected to tDCS, 46 participants were given sentences for passive reading. Sentences were displayed in segments with a fixed time of exposition, and a verb, either the one mentioned in the sentence or an alternative one was displayed 1,500 ms after the sentence (e.g., included vs. excluded, in the example). Participants were told to read them and then press the space bar to continue the experiment. Results showed shorter latencies for approach verbs that were either mentioned in approach sentences or the alternatives in avoidance sentences, both in affirmative and negative versions under anodal conditions compared to sham conditions. Thus, the anodal stimulation of rSTS affected the accessibility of approach verbs that were not modulated either by being mentioned or by sentence polarity. In addition, mentioned verbs had shorter reading times than the alternative ones in negative sentences in the anodal vs. sham condition. This suggests that stimulation caused an effect of negation in the activation of the mentioned verb. Implications are discussed in the context of the role of the rSTS in processing attitudinal verbs and negation to understand better approach and avoidance mediated by language in the framework of the two-step model of negation processing.

## Introduction

Language allows us to describe how we affectively interact with environmental stimuli providing an indication of individuals’ attitudes, either pro (approach) or against (avoidance), to environmental stimuli in different contexts by means of verbs, such as accept vs. reject, praise vs. despise, approve vs. criticize, and so on. The purpose of these verbs could be the description of approach-avoidance dynamics either toward other people, e.g., “include/exclude someone in/from a group of friends”; or objects (e.g., choose or reject a book), or things on a more abstract level (e.g., choose or reject dance as entertainment). In this way, and by means of experiential simulation (Zwaan, 2004), language facilitates the representation and communication of peoples’ attitudes (likes or dislikes) and intentionality in social life (Marrero et al., 2015, 2017, 2019, 2020a,^2022^).

Intentionality processing has been associated with the recruitment of the temporal lobe (anterior temporal lobe, superior temporal sulcus, middle and superior temporal gyrus) and the precuneus and temporo-parietal junction that constitute a “mentalizing” network (Spunt et al., 2010; Dodell-Feder et al., 2011; Kennedy and Adolphs, 2012). It is relevant to distinguish between the representation of intentions as mental states not associated with current actions and the representation of intentions and goals that are inherent in perceived actions. The latter involves a neural system particularly associated with the superior temporal sulcus (STS) and is recruited for action understanding (Gobbini et al., 2007; Spunt et al., 2010). Moreover, the activation of this mentalizing network to process social information is usually stronger in the right hemisphere (Wong and Gallate, 2012; Watson et al., 2014).

According to Spunt et al. (2010), there are two natural modes of representing human social actions (e.g., lifting weights, or brushing teeth): the “How” and the “Why” the action is performed. The Why (e.g., to be stronger in the case of lifting weights) involves goals and intentionality that typically recruit the mentalizing network. In this context, approach and avoidance attitudinal actions would be implicitly why actions. Attitudinal verbs are relatively abstract as they describe intentionality in our interactions with environmental targets, rather than specific action patterns. For example, the utterance “she excluded meat from her diet” implicitly describes against attitude (avoidance) to meat intake as the why for this action, but not the specific action, which might be instantiated in a variety of ways: i.e., asking for vegetarian dishes in restaurants. Social information conveyed by verbs and sentences is about the protagonist’s preferences and aversions. This is important for verbal social communication. For example, nobody cooks meat in a romantic dinner encounter if they have heard the other person has excluded meat from their diet (e.g., they are vegetarian). In this way, language facilitates interactions for social relationships.

In this regard, it has been shown that approach/avoidance intentionality recruits the mentalizing network, and particularly the rSTS either with objects (Vander Wyk et al., 2009) or persons in social perception with greater activation in approach than avoidance (Pelphrey and Morris, 2006; Pelphrey and Carter, 2008; Saitovitch et al., 2012; Johnson et al., 2015; Yang et al., 2015), and also in mutual liking (Flores et al., 2018). Likewise, Ross and Olson (2010) (see also Tavares et al., 2008), using a version of the Heider and Simmel animation task in an fMRI study, reported the activation of more anterior aspects of the rSTS when participants judged “friendship” from simple geometric shape interactions. Similarly, Gobbini et al. (2007) have reported activation along the full length of the rSTS when participants observed Heider and Simmel animations and made social intentional judgments of interactions. Thus, previous research has shown activation of the right brain temporal area (around STS, middle aspects) in processing approaches and avoidance in social perception with images. However, as far as we know, the role of this brain area in processing approach and avoidance verbally described has not been examined.

Previous research in brain processing of approach and avoidance attitudinal sentences has shown that anodal tDCS stimulation (excitation) of middle to anterior aspects of rSTS enhances the memorization of approach compared to avoidance in the context of social relationships (Marrero et al., 2020a), and facilitates approach sentences in a reading task modulated by approach and avoidance personality traits (Reyes et al., 2021). Thus, approach attitudinal verbs seem to benefit from tDCS stimulation of the rSTS, which agrees with previous research that supports the specialization of the rSTS for approach processing.

In this study, we examine, for the first time, whether tDCS stimulation of the rSTS improves the processing of attitudinal verbs in the context of affirmative and negative sentences. Negation would constitute a linguistic operator (see Horn, 1989) at the service of social communication. Indeed, in the case of attitudinal expressions negation reverses the direction from approach to avoidance (e.g., not include to exclude) and vice versa from avoidance to approach (e.g., not exclude to include), which significantly enriches the communication of our preferences and aversions in social life (Marrero et al., 2020b). We consider it to be theoretically relevant to include sentential negation in our study for a more in-depth examination of the type of effect of tDCS on the rSTS on attitudinal verb processing. The so-called two-step model (Kaup, 2006; Kaup et al., 2006; Dudschig and Kaup, 2018), which is based on the more general embodied simulation theory (EST, Zwaan, 2004; Barsalou, 2008), separates the negative sentence comprehension into two sequential steps. First, the negated situation (e.g., a “closed door” for the sentence *The door is not closed*) is represented, and a second step where this state of affairs is rejected. In the first step, the negated information is active, whereas, in the second step, it is inhibited (this does not happen). In the second step, implications about alternatives to the rejected state of affairs (e.g., an “open door”) could be triggered and the alternative is represented. This is predicted when sentence content enables the representation of an alternative state of affairs (Kaup et al., 2006; Dudschig and Kaup, 2018), e.g., an “open door” for “the door is not closed,” or when it involves binary categories as could be the case of approach/avoidance verbs, e.g., negation of approach in this example “they did not exclude meat in their diet” would trigger the alternative state of affairs representation “meat included” (Marrero et al., 2020b).

Although the role of anodal stimulation has been well-established by previous research, the effect of cathodal stimulation is less clear, and sometimes results in task performance enhancement (Dedoncker et al., 2016). Thus, in this study, we contrast anodal vs. sham tDCS conditions. In light of previous research, we predict that anodal stimulation will affect the encoding of approach meaning. Consequently, an effect of anodal stimulation in the rSTS in the integration of negation in the sentence meaning could be expected. In this case, for approach meaning, we predict greater availability (shorter verb reading times) in approach verbs in affirmative sentences when they have been mentioned, and in approach verbs in negative avoidance sentences when they are alternative verbs (e.g., the sentence: “Julio did not exclude meat from the grocery list” is followed by the verb “included” as the target to be read). This would occur online during understanding if time is given to integrating the negation in the sentence meaning, with a delay of 1,500 ms between the sentence reading and the display of the word target for recognition (see Kaup, 2006).

## Materials and methods

### Participants

We recruited 47 university students (16 male participants, mean age 20 ± 2 years standard deviation) from the University of La Laguna without a history of neurological or psychiatric diseases. Twenty-three participants were randomly assigned to the anodal stimulation condition, and 24 participants to the sham (placebo) condition. The sample size was calculated using the effects found in Marrero et al. (2020b) for a small-medium effect size with a power of 0.8, with a *p*-value of 0.05.

The study was approved by the University of La Laguna’s Committee on Ethics in Research and Animal Welfare (CEIBA2017-0272) as part of a research project (2018–2021) funded by the Spanish Government: PSI2017-84527-P. All the participants fulfilled the inclusion criteria for non-invasive electrical brain stimulation (Bikson et al., 2016): no epilepsy (or close relatives), no migraines, no brain damage or head injuries, no metal parts and/or pacemakers, no drugs that could alter brain activity, and being right-handed according to the Edinburgh Handedness Inventory (Oldfield, 1971). They received a monetary incentive of 10 euros in exchange for participating in the experiment.

### Materials and stimuli

The experimental sentences had previously been subjected to normative studies on the motivational direction of the sentences (approach-avoidance), controlling for linguistic factors such as sentence length, target length, and number of syllables, as well as psycholinguistic factors such as imaginability (see Marrero et al., 2020b). One hundred and forty sentences, 10 per experimental condition and 60 filler sentences were used in this study. Filler sentences had also approach and avoidance contents but they had not been experimentally manipulated and were aimed at varying and widening sentence social contexts and contents on reading in the conjoint of sentences. Examples of the experimental sentences are shown in Table 1.

**TABLE 1:** Example of approach and avoidance sentences in affirmative and negative versions.

Approach	Avoidance
Julio/(did not)	Julio/**(did not)**
include/meat/on/the/grocery/list)	**exclude**/meat/on/the/grocery/list)

### Design and procedure

A 2 × 2 × 2 × 2 factorial design was used, with Direction (approach and avoidance) × Polarity (affirmative vs. negative) × Verb (alternative vs. mentioned) as within-subjects factors and stimulation (anodal vs. sham) as a between-subjects factor. The dependent variable was the reading latency of the target verb.

Once participants arrived at the laboratory, they were given a personal data form and a questionnaire to screen for exclusion conditions, and they signed an informed consent form. Participants were told that the experiment consisted of performing a reading task on the computer, followed by non-invasive electrical stimulation (tDCS), and then returning to the reading task on the computer. They had no information regarding which tDCS condition they had been assigned to. No direct assessment of blinding was performed.

Participants were told to read sentences that appeared while seated in front of a computer screen. First, they received instructions for carrying out the task and a training task with eight sentences. After completing this training, and before the application of tDCS, they were presented with a group of 30 sentences under different conditions in order to have a general measure of differences in sentence reading time between anodal and sham participants before stimulation. Subsequently, participants were given 20 min of anodal tDCS or placebo stimulation in the sham condition. Following tDCS, they performed the experimental task.

Each sentence presentation started with a cross point displayed in the middle of the screen for 750 ms. Following an interval of 150 ms, one sentence was displayed. Sentence presentation was segmented as in the following example:

“*Petra/aceptó/el recibo/del/banco/de la/localidad*” (“Petra/accepted/the receipt/of the/bank/of the/town”); in the negative version: “*Petra/no aceptó/el recibo/del/banco/de la/localidad*” (“Petra/did not accept/the receipt/of the/bank/of the/town”). Each segment was displayed for 300 ms with an interval of 150 ms between them.

After the sentence was displayed, participants were presented with a word (either the verb mentioned in the sentence or the alternative verb, “accepted” vs. “rejected” in the previous example). This word appeared 1,500 ms after the sentence display ended. Participants were instructed to read the verb and then press the space bar (indicated on the keyboard) to proceed. The word remained on the screen for 3,000 ms, or until a response was received. The interval between each sentence display was 750 ms. Participants were given 140 sentences, 10 for each experimental condition and 60 filler sentences. Filler sentences were thematically similar to experimental sentences with affirmative and negative versions. In this way, participants read a greater variety of verbal actions and contexts.

To prevent participants from focusing exclusively on the superficial reading of the target verb, one-quarter of the sentences (36) were immediately followed by a question about the content just read (e.g., “Is it stated that Petra rejected the receipt of the bank?”). This question had either a positive (YES, indicated on the keyboard over the letter “P”) or negative (NO, indicated on the keyboard over the letter “Q”) response half of the time and remained on the screen for 5,000 ms or until a response was made. Feedback was given to the participants and displayed for 2,000 ms. After a delay of 750 ms, a new sentence was displayed.

Participants were randomly assigned to one of the sets of sentences resulting from counterbalancing experimental conditions. This ensured that every participant received an equal number of sentences for each of the four conditions, and no participant received the same sentence two times. Sentences were randomly presented to the participants in each counterbalanced set. At the end of the session, they were thanked, and a brief explanation of the experiment was given to them for debriefing. Moreover, they were asked not to discuss the experiment with other potential participants. The experimental session lasted around 60 min.

### Transcranial direct current stimulation in the right superior temporal sulcus

Transcranial direct current stimulation (tDCS) is a non-invasive brain stimulation tool that has shown great potential in improving cognitive performance. In this regard, there is evidence showing tDCS as a tool that helps us to better understand the cortical substrates that underlie cognitive functions (Filmer et al., 2014). tDCS uses weak and constant electrical currents (typically up to 2 mA) to induce short-term changes in the excitability and cortical activation of the brain regions we wish to stimulate. Two effects can be generated, excitation or inhibition, and this depends on the polarity of the current. If we use anodal tDCS, it increases the probability of firing action potentials through the depolarization of neuronal membranes altering spontaneous brain activity and increasing the activity of the brain area below the anode (Stagg et al., 2018). This leads to an improvement in cognitive functions associated with the stimulated brain region as well as brain areas that are functionally linked. Therefore, tDCS offers us the possibility to establish causal relationships between cognitive function and the cortical structure with its network.

**FIGURE 1:**
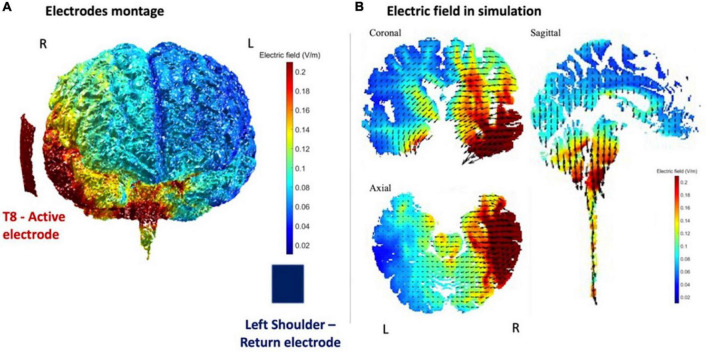
ROAST (realistic volumetric approach to simulate transcranial electric stimulation, Huang et al., 2019) simulation of electric field magnitude in T8 anode (active electrode) and in the place nearest to the contralateral shoulder cathode (return electrode) stimulation setup. **(A)** Electrode montage and **(B)** electric field magnitude simulation in axial, coronal, and sagittal planes. Taken from Reyes et al. (2021).

CE-certified battery-powered stimulator (neuroConn DC-STIMULATOR. neuroConn GmbH, Albert-Einstein-Str. 3, 98693 Ilmenau, Germany) was used for the non-invasive tDCS current conduction with an intensity of 2 mA. The size of the rubber electrodes was 5 × 5 cm (active electrode) and 7 × 5 cm (return electrode), which resulted in a density of 0.08 and 0.057 mA/cm^2^, respectively. Both were covered with sponges soaked in saline. The active electrode was placed on the scalp in accordance with the international 10–20 EEG system. The montage used for tDCS was anode over T8 and return electrode over the contralateral shoulder (Bikson et al., 2010). We stimulated BA 22 and BA 21 brain areas overlapping medial aspects of rSTS, as shown in Figure 1. This stimulated area is a part of the mentalizing network (Spunt et al., 2010; Dodell-Feder et al., 2011; Kennedy and Adolphs, 2012), which is specialized in processing social intentionality. The stimulation application time was established based on previous studies of tDCS (Nitsche and Paulus, 2001). We used 20 min with 15 s ramp-up and ramp-down periods. During the false tDCS (sham) condition, participants followed the same procedure as active stimulation and with the same electrode montage. The only difference was that sham stimulation lasted only 45 s (ramp-up: 15 s maximum intensity, and ramp-down 15 s). During data collection, a counterbalance was performed in which active stimulation and sham stimulation were alternated.

### Transcranial direct current stimulation procedure

Participants performed the first block of sentences of the reading task. Once the first block was finished, the electrodes were placed on the participant and tDCS started following the tDCS protocol. Right after the removal of the tDCS equipment, participants performed the block of experimental sentences. The stimulation parameters were considered safe (Bikson et al., 2016). We asked participants to inform us of any adverse effects during tDCS (Brunoni et al., 2011; Kessler et al., 2012).

## Results

During the intervention, participants reported mild and transient adverse effects. Table 2 shows the type of adverse effect, the severity, and the percentage of the participants who experienced it.

**TABLE 2:** Adverse effects, severity, and rounded percentage of participants that experienced them in the tDCS study.

Type of effect	Severity	% Anodal	% Sham
Tingling	Mild	18	10
Itching	Mild	62	43
Burning sensation	Mild	09	05
Discomfort	Mild	06	04

Following Reyes et al. (2021), reading latencies above/under 2.5 SD of the participant mean (14.61%) were removed from the analysis. One participant was removed in the anodal condition because three conditions had no data as a result of removing extreme reading latencies. Participants’ mean latencies over 2 *SD* of the group mean in each condition were substituted for the group mean in the condition (1.76%). A comparison of means of verb reading latencies before tDCS stimulation was performed and no significant differences were found between anodal and sham groups, *p* > 0.10.

Repeated measures ANOVA were conducted with three within-subjects factors: sentence direction (approach vs. avoidance), polarity (affirmative vs. negative), and verb (mentioned vs. alternative), and one between-subject factor, tDCS stimulation: anodal and sham. The direction × verb interaction was found to be significant, *F*(1, 44) = 6.13, *p* = 0.017, *η_*p*_^2^* = 0.122. As shown in Table 3, the mentioned verbs in approach sentences had shorter reading times than the alternative verbs: *Mean Diff.* = 30.60, *SD* = 76.19, *t*(45) = 2.72, *p* = 0.009. Likewise, the alternative verbs had shorter reading times in avoidance than in approach sentences: *Mean Diff.* = 22.80, *SD* = 70.76, *t*(45) = 2.18, *p* = 0.034. Considering that alternative verbs in avoidance sentences are approach verbs, this result supports the reading advantage of approach verbs compared to avoidance verbs.

**TABLE 3:** Means and standard deviations of reading time (ms) of mentioned and alternative verbs in approach and avoidance sentences.

Sentence direction	Verb	Mean	*SD*
Approach	Mentioned	595.48	142.86
	Alternative	626.08	150.08
Avoidance	Alternative	606.22	138.51
	Mentioned	603.26	152.40

This interaction was qualified by the interaction direction × verb × tDCS, which was significant, *F*(1, 44) = 4.45, *p* = 0.041, *η_*p*_^2^* = 0.092 (see Table 4). Follow-up comparisons showed that in the anodal condition, reading times of mentioned verbs were significantly shorter than that of the alternative verbs in approach sentences: *Mean Diff.* = 57.71, *SD* = 93.89, *t*(21) = 2.88, *p* = 0.009. By contrast, reading latencies of alternative verbs were significantly shorter in avoidance sentences than in approach sentences: *Mean Diff*. = 37.82, *SD* = 85.09, *t*(21) = 2.08, *p* = 0.049. However, neither were significant under the sham condition (*p* > 0.10). This interaction showed that shorter reading times for approach vs. avoidance verbs are associated with anodal condition.

**TABLE 4:** Means and standard deviations of reading time (ms) of mentioned and alternative verbs in approach and avoidance sentences under each tDCS condition.

Direction	Verb	Mean	*SD*
**(a) Anodal**			
Approach	Mentioned	558.48	130.95
	Alternative	616.20	148.42
Avoidance	Mentioned	585.17	132.73
	Alternative	578.38	141.58
**(b) Sham**			
Approach	Mentioned	629.39	147.52
	Alternative	635.14	154.21
Avoidance	Mentioned	625.51	143.67
	Alternative	626.11	161.26

The polarity × verb interaction was significant, *F*(1, 44) = 5.51, *p* = 0.023, *η_*p*_^2^* = 0.111 (see Table 5). Follow-up comparisons showed that in the negative sentences, mentioned verbs had shorter reading times than the alternative verbs: *Mean Diff.* = 28.83, *SD* = 79.51, *t*(45) = 2.45, *p* = 0.018. Likewise, mentioned verbs had shorter reading times in negative than in affirmative sentences: *Mean Diff.* = 18.53, *SD* = 61.66, *t*(45) = 2.07, *p* = 0.044. These results support that reading latencies of mentioned verbs (in comparison to the alternatives) improved in negative sentences compared to affirmative sentences.

**TABLE 5:** Means and standard deviations of reading time (ms) of mentioned and alternative verbs in affirmative and negative sentences.

Polarity	Verb	Mean	*SD*
Affirmative	Mentioned	610.22	131.60
	Alternative	609.10	148.38
Negative	Mentioned	591.43	148.87
	Alternative	620.26	153.26

The interaction was qualified by the interaction polarity × verb × tDCS, *F*(2, 44) = 4.08, *p* = 0.049, *η_*p*_^2^* = 0.085 (see Table 6). Follow-up comparisons showed that in the anodal condition the reading times in negative sentences of mentioned verbs were significantly shorter in comparison with: alternative verbs in the negative sentences, *Mean Diff.* = 54.44, *SD* = 78.08, *t*(21) = 3.27, *p* = 0.004; alternative verbs in affirmative sentences, *Mean Diff.* = 37.34, *SD* = 83.50, *t*(21) = 2.09, *p* = 0.048; and mentioned verbs in affirmative sentences, *Mean Diff.* = 40.86, *SD* = 72.49, *t*(21) = 2.64, *p* = 0.015. In the sham condition, neither was significant. This interaction showed that the shorter reading time of mentioned verbs in negative sentences is associated with the anodal condition. No main effects of Verb, Direction, and Polarity, and tDCS neither of interactions verb × direction, verb × direction × polarity, and verb × direction × polarity × tDCS were found.

**TABLE 6:** Means and standard deviations of reading time (ms) of mentioned and alternative verbs in affirmative and negative sentences of each tDCS condition.

Polarity	Verb	Mean	*SD*
**(a) Anodal**			
Affirmative	Mentioned	592.26	131.87
	Alternative	588.74	136.35
Negative	Mentioned	551.39	133.12
	Alternative	605.84	146.40
**(b) Sham**			
Affirmative	Mentioned	626.78	131.95
	Alternative	627.77	159.19
Negative	Mentioned	628.13	155.70
	Alternative	633.48	161.25

## Discussion

In this study, we examined the effect of tDCS stimulation on the rSTS in reading times of approach/avoidance attitudinal verbs in the context of affirmative and negative sentences. We expected an effect of anodal stimulation vs. sham on the processing approach in comparison with avoidance meaning in the accessibility of approach verbs. Thus, in the anodal condition, we predicted that reading time would improve when approach verbs were mentioned in affirmative sentences and, more importantly, when they were the alternative in negative avoidance sentences. Contrary to our predictions, there was an effect on approach verb accessibility compared to avoidance verbs either mentioned or alternative and in affirmative or negative sentences.

We also found an effect of the interaction polarity × verb × tDCS. Mentioned verbs had shorter reading times than alternative ones in negative sentences than in affirmative sentences in the anodal condition, in contrast to the sham condition. In accordance with the two-step model, a facilitatory (or at least not inhibitory) effect of negation on the availability of negated information occurs at the first step: at the start of negation processing for sentence understanding. At this moment, negation is still not fully processed, and the focus is on representing the negated information for sentence understanding. Thus, it seems that the anodal stimulation of the rSTS enhances the role of negation by keeping the negated verb activated in the first step. This would delay the process of integrating negation in sentence meaning in the second step, which could explain why anodal stimulation had no effect on approach meaning (the case of the alternative verb in negative avoidance sentences). In accordance with the two-step model (Kaup, 2006), the effect of tDCS on the rSTS on negation suggests that this brain area could be involved in the first step where negation puts the focus on processing the negated information.

Our study suggests that the anodal stimulation of the rSTS affects the general availability of approach verbs that are not modulated by the meaning of the previously read sentence. However, the effect of polarity in verb reading time supports that in the anodal condition, negation is being processed for sentence understanding. Thus, assuming that participants process sentences for understanding, we consider an alternative explanation of the effect of anodal stimulation in approach verbs. In accordance with previous research, avoidance verbs would be represented as “not approach” and thus involve an implicit negation (see Marrero et al., 2020b). In this case, implicit negation, like explicit negation, may focus on what is negated: the “approach verb” resulting in greater activation and shorter reading times when approach verbs are the alternative in avoidance sentences. Further research is thus necessary to examine negation processing and avoidance encoding by considering avoidance verbs as implicitly negative.

### Contributions and future implications

Understanding attitudes and intentionality of human actions recruit the Mentalizing Network (Gobbini et al., 2007; Spunt et al., 2010; Dodell-Feder et al., 2011; Kennedy and Adolphs, 2012), which includes the superior temporal area, around the rSTS. Beyond the role of the rSTS in processing approach attitudes in social perception (e.g., mutual vs. averted gaze) (Pelphrey and Morris, 2006; Pelphrey and Carter, 2008; Saitovitch et al., 2012; Johnson et al., 2015; Yang et al., 2015) and with the Heider and Simmel task (Gobbini et al., 2007; Tavares et al., 2008; Ross and Olson, 2010), our study has shown that approach and avoidance are also represented by language and demonstrates that rSTS stimulation improves the processing of approach attitudinal verbs.

Likewise, our study suggests, for the first time, that the rSTS would modulate the brain processing of negation following the two-step model of negation processing and of avoidance verbs, as they involve an implicit negation. Stimulation of the rSTS seems to cause activation of what is negated in the first step of negation processing in accordance with the two-step model (Kaup, 2006).

### Limitations

For the anatomical localization of the STS, we considered the position of electrode T8 of the EEG montage; however, aspects such as the anatomical variability across subjects and the lack of the focality of the stimulation that was applied might have played a role in the results. Therefore, future research is necessary to confirm the role of the brain temporal area around rSTS (middle aspects) in processing approach in attitudinal sentences by considering the use of other techniques such as fMRI or TMS that could enable more direct and precise evidence of its potential role. Moreover, our study’s participants were young university students with a predominant presence of female participants. Nevertheless, approach and avoidance brain encoding could be affected by developmental changes or modulated by gender. Hence, future studies should include a broader age range and more male participants.

Moreover, non-social situations have not been included as a control in our study. Thus, it could be that stimulation would also affect language comprehension in general, regardless of content. However, the effect found of anodal stimulation benefitting approach verb reading time seems to counteract the explanation of a general effect of stimulation. Another limitation of our research is the fact that our task was an easy task: passive reading of verb targets. More cognitive demanding recognition tasks (e.g., probe-word naming) could counteract an effect of motivation in the placebo condition in order to show whether negation is integrated into meaning after 1,500 ms following sentence reading in verb availability. Likewise, it could have been interesting to analyze the moderator role of gender in the stimulation effect, as gender cultural and educational factors may influence social and affective contents. However, the number of male participants was not sufficient to carry out an analysis. Future research is then necessary to examine the moderator role of gender in the effect of stimulation on the right brain temporal area in processing approach and avoidance and negation verbally described.

## Conclusion

In this study, we examine the effect of anodal tDCS on the rSTS in the processing approach and avoidance in attitudinal verbs with negation. Our results show that anodal stimulation improves the availability of approach verbs with no facilitation of the integration of negation in sentence meaning. In addition, anodal stimulation benefited verbs when mentioned in negative sentences. We interpret these results within the framework of the two-step model of negation processing. Stimulation of the rSTS could affect negation processing by activating what is negated in the first step. As avoidance verbs involve an implicit negation (as they would be represented as no-approach), approach verbs would be first activated for processing avoidance verbs. Thus, the rSTS (and temporal areas around it) processes approach and causes the activation of what is negated either explicitly or implicitly as in avoidance verbs.

## Data availability statement

The raw data supporting the conclusions of this article will be made available by the authors, without undue reservation.

## Ethics statement

This study was conducted according to the guidelines of the Declaration of Helsinki, and approved by the Ethics Committee of University of La Laguna (CEIBA 2017–0272, December, 22, 2017). The patients/participants provided their written informed consent to participate in this study.

## Author contributions

AN and IP were responsible for the design and interpretation of data, drafting the article, and revising it critically for important intellectual content. CR-M was responsible for the collection and analysis of data. HM was responsible for the conception, design, analysis interpretation of data, and for revising the article critically for important intellectual content. All authors contributed to the article and approved the submitted version.
